# Correction: The evolution of bone-eating worm diversity in the Upper Cretaceous Chalk Group of the United Kingdom

**DOI:** 10.1371/journal.pone.0326451

**Published:** 2025-06-13

**Authors:** Sarah Jamison-Todd, James D. Witts, Marc E. H. Jones, Deborah Tangunan, Kim Chandler, Paul Bown, Richard J. Twitchett

The systematic ichnotaxonomy section in this article was incomplete [1], as it omitted the Life Science Identifiers (LSIDs) for the seven new ichnospecies. To comply with the amended International Code of Zoological Nomenclature for electronic publications [2,3], the LSIDS are provided here as part of a corrected systematic ichnotaxonomy section. The authors also provide more detailed nomenclatural justifications for each ichnospecies. The LSID for this publication is: urn:lsid:zoobank.org:pub:DD81124D-44C1-41E1-9833-408F7D3AE4E0.

Please see the updated Systematic ichnotaxonomy below.

## Systematic ichnotaxonomy

*Osspecus* igen. Higgs et al. 2012 [4]

Holotype:

Boring number 1 (Figs 3(a) and 4a–c) in [4] in a fossil cetacean radius stored in the Museo di Storia Naturale, Sezione di Geologia e Paleontologia, Florence, Italy (IGF 1134T).

**Fig 3:**
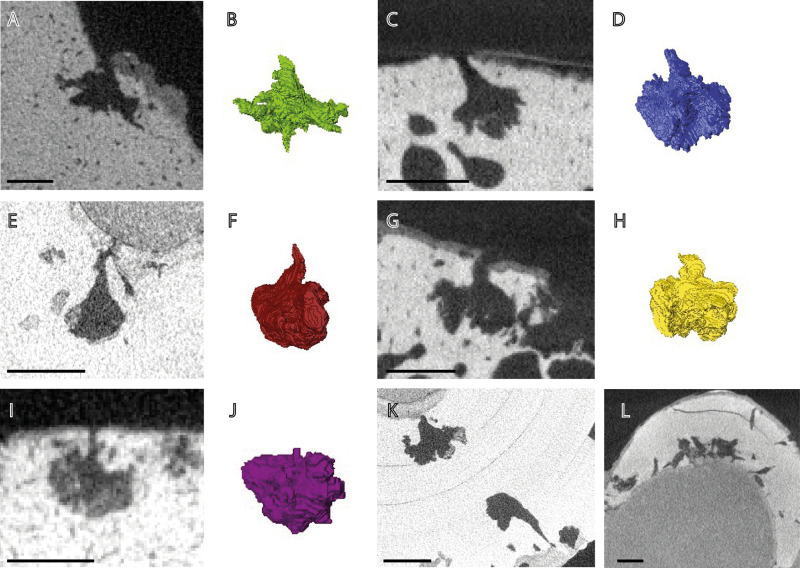
The 2D cross-sections and 3D reconstructions of the type borings of each of the five morphotypes present in the specimens addressed in this study. A) Cross-section of an *O. eunicefooteae* boring in NHMUK PV OR 32812. B) Three-dimensional reconstruction of the boring in panel A. C) Cross-section of an *O. tuscia* boring NHMUK PV R 1233. D) Three-dimensional reconstruction of the boring in panel C. E) Cross-section of an *O. morus* boring in NHMUK PV R 1215. F) Three-dimensional reconstruction of the boring in panel E. G) Cross-section of an *O. campanicum* boring in NHMUK PV R 1233. H) Three-dimensional reconstruction of the type boring in panel G. I) Cross-section of an *O. arboreum* boring in NHMUK PV R 4205. J) Three-dimensional reconstruction of the boring in panel I. K) Different morphologies of *O. morus* borings in NHMUK PV R 1215. L) Tubular bioerosion in NHMUK PV R 1265 that may be exploratory and incomplete *Osedax* borings or another form of bioerosion. Scale bars are 2 mm in all panels.

**Fig 4:**
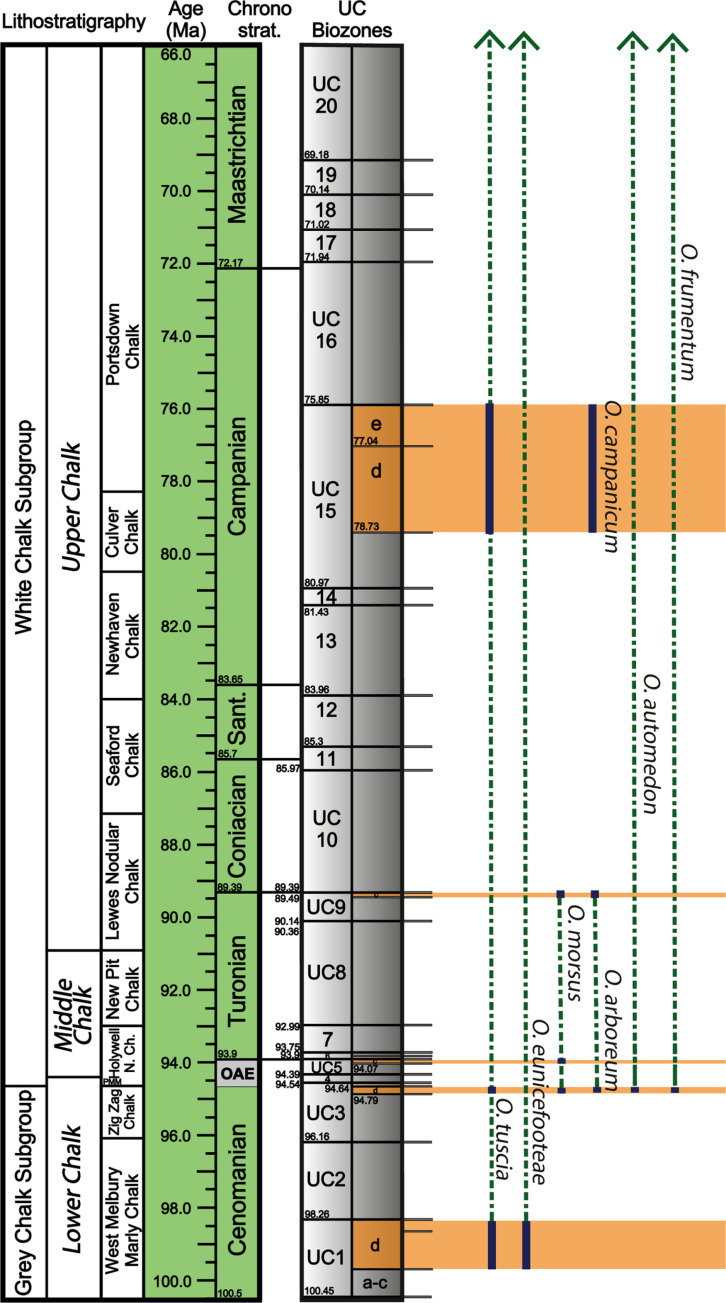
Litho- and biostratigraphic scheme for the UK Chalk Group, showing the succession of calcareous nannofossil zones, and current lithostratigraphic divisions. Constrained marine reptile specimen dates are marked by orange bars. Ichnospecies occurrences in the Upper Cretaceous are indicated by navy bars, and inferred ranges by dashed green lines. Ichnospecies that are found in the Cenozoic have an inferred range above the Cretaceous, indicated by arrows. Nannofossil zonation scheme is modified from Burnett (1998) [12] and lithostratigraphy is modified from Rawson et al. (2001) [13]. Note that *O. panatlanticum* is not found in the UK Chalk. Numerical ages from GTS2020 [14] and TimescaleCreator8 (timescalecreator.org).

Diagnosis:

Single entry borings found in bone substrates. Individual borings consist of a circular to sub-circular aperture, lacking any rim or platform. This aperture extends into the bone as a uniformly thick canal, generally perpendicular to the bone surface, with a globular or irregularly shaped chamber at the base of the canal. These chambers may or may not have thin exploratory tunnels emanating from them.

*Osspecus tuscia* isp. Higgs et al. 2012 [4]

Holotype:

Same as genus

Diagnosis [4]:

Boring with sub-millimetre-sized apertures. The base of the apertural canal tapers into a chamber that is partially flattened in the vertical plane. Short globular exploratory lobes extend from the main body of the chamber.

Emended diagnosis:

Boring with chamber diameters of 0.7–2.0 mm and total depth of 0.6–2.1 mm. The wide aperture leads to a tapering aperture neck that is wider at the base near the top of the chamber. The chamber sits at mid-depth below the surface of the bone. Radial symmetry is centred at the base of the aperture neck, and no secondary symmetry is evident. Branches are of mid-length relative to the chamber and maximum arc length is 180 degrees or less, so that the branches generally point outwards or inwards but not up towards the bone surface. Branch shape is lobate and also tapering, with branches having pointed ends and widening into the chamber.

Remarks:

The measured borings from Higgs et al. (2012) [4] are larger, but they overlap in size ranges with those measured for this study. The lesser arc length and tapering nature of the branches and aperture neck gives the borings the appearance of being slightly vertically stretched, but some of the borings in Higgs et al., though sharing the other features that define this morphotype, are vertically flattened. The original description as it stands therefore encompasses some variant morphologies. Here, the more vertically stretched endmember with a shorter arc length is predominant. This ichnospecies is present in three marine reptile specimens. Figured here in Fig 3, panels C and D.

*Osspecus eunicefooteae* isp. nov.

LSID urn:lsid:zoobank.org:act:7067E5BC-4DF3-4278-B14D-4B9051372BF6

Etymology

In honour of Eunice Newton Foote (1819–1888), the first person to suggest on experimental grounds that an increase in atmospheric CO2 would in turn increase the temperature of the Earth [5,6]. She was also an inventor and campaigned for women’s rights [6]. This species name consists of the name Eunice Foote in the genitive case. This ichnospecies was originally intended to be called *Osspecus eunicefootia* [1], with *eunicefootia* used as a noun in apposition to the genus. The unconventional but code-compliant -ia ending was chosen to mirror that of the type species *O. tuscia*. On reflection, we have instead chosen *eunicefooteae* as a noun in the genitive case to better reflect the recommendations of the ICZN article 11.9 [7].

Holotype:

NHMUK PX TF 309

Fig 3 this study, panels A and B, boring in NHMUK PV OR 32812

Additional examples:

Jamison-Todd et al. (2024) [8], Fig 2, panels G and H.

Higgs et al. (2014) [4], Fig 3.

Diagnosis:

Boring with chamber diameters of 1.8–2.7 mm and total depths of 1.2–1.9 mm. Apertures are wide relative to other boring types. Borings are shallow, the main chamber sitting just below the surface of the bone, with a short aperture neck. The centre of radial symmetry sits at the top of the chamber. Branches are wavy, thin, and long relative to the chamber, though they can be of irregular length. Overall chamber shape is approximately hemispherical, with an arc length generally close to 180 degrees.

Remarks:

The chambers are large relative to most other *Osspecus* ichnospecies. Due to the shallow depth of the hemispherical chambers they are prone to collapse and weathering, with the interior of the chamber exposed and the outer layer of bone covering the boring unpreserved. These borings are found in one marine reptile specimen in this study, and were previously described in Jamison-Todd et al. (2024) in a plesiosaur from North America, which represents a particularly large example of this boring type [8]. This morphotype was therefore present on both sides of the Atlantic Ocean basin in the Cretaceous. The living species *Osedax antarcticus* also creates borings that can be referred to this new ichnospecies [8].

*Osspecus morsus* isp. nov.

LSID urn:lsid:zoobank.org:act:042D5EB4-49AB-421E-A40C-6166D037B4B1

Etymology:

After Latin for ‘bite’, in double reference to the eating of the bones and the fact this boring type is usually found in teeth. ‘Morsus’ is used here as a noun and not as a verb.

Holotype:

NHMUK PX TF 310

Fig 3 this study, panels E and F. Boring in NHMUK PV R 1215.

Additional examples:

Jamison-Todd et al. (2024) [8], Fig 2, panels C and D.

Diagnosis:

Borings with chamber diameters ranging from 1.3–2.4 mm, and total depths from 2.3–3.7 mm. Aperture necks can be variable in length. Chambers that are set deeper within the bone are connected to long, curved aperture necks leading to proportionally small chambers. The centre of radial symmetry of individual chambers is set at the base of the aperture neck, and there are commonly clusters of branches within these chambers, with individual radial symmetry. Branches are small and lobate and can create a ridged appearance around the edge of the chamber. Arc length is also variable, with some chambers branching widely over 180 degrees and others branching in a narrow minor arc of less than 90 degrees, giving the chambers a triangular cross-sectional shape that sometimes spreads into a broader arc.

Remarks:

This morphotype is the most variable in appearance and broad morphology. Some of the variability in this boring morphotype is figured here in Fig 3, panel K. This type of boring is commonly but not always found in the dentine at the base of teeth, occasionally puncturing the enamelled areas. The boring depth and the curvature of the aperture necks may be related to density differences within the dentine, but the histology of dentine is poorly documented in reptiles. It is also possible that the morphological variability of this boring type may represent the borings of multiple species. It is also not clear whether the clusters of chambers are made by the same animal as a result of secondary symmetry in the morphology of the worm, or if multiple animals are exploiting the same point of entry into the bone. The commonalities between certain features of these borings, however, lead to their classification into one type. Borings previously described in Jamison-Todd et al. (2024) as ‘type 2’ also belong to this ichnospecies and co-occur in one tooth with two other ichnospecies [8]. These borings are present in two of the specimens presented here.

*Osspecus campanicum* isp. nov.

LSID urn:lsid:zoobank.org:act:3C1223FC-8618-4964-A77C-AF52C14DD12E

Etymology:

This is the only ichnospecies found exclusively in the Campanian Stage of the Late Cretaceous. The species name is a Latinization of the proper noun of the geological stage.

Holotype:

NHMUK PX TF 311

Fig 3 this study, panels G and H. Boring in NHMUK PV R 1233.

Diagnosis:

Boring with chamber diameters ranging from 0.5–1.4 mm, and total depth of 0.5–2.0 mm. Apertures are wide and the aperture necks leading into the chambers are columnar. The chambers sit at mid-depth in the bone. The centre of symmetry is not always distinct, though it generally sits towards the upper middle of the chamber. Symmetry is not regularly radial, with the branches pointing mostly inwards, and the presence of secondary symmetry in some clusters of branches is possible. Chambers are therefore irregularly shaped, and the branches are not of equal size and length. Branch shape is lobate and rounded. Arc length is approximately 180 degrees.

Remarks:

The irregular rounded branches give this boring type a squat cartoonish appearance. Smaller borings may make clusters that create larger pits when they are collapsed or weathered. These borings share some features with *O. tuscia*, and are of a similar scale, the main differences in *O. campanicum* being the wider aperture and the rounded shape of the lobate branches; neither aperture nor branches have a tapering appearance as in *O. tuscia* [4]. This morphotype is found here in one specimen.

*Osspecus arboreum* isp. nov.

LSID urn:lsid:zoobank.org:act:64FC9755-3A80-4D7B-BF8D-5D100439BE08

Etymology:

For its resemblance to a typical tree shape. In clusters the borings resemble tiny forests in cross section. For adjectival species names such as this one, we take the secondary poetic usage of the Greek ‘specus’ as neuter, as the original genus was never defined in gender, and Latinized species names therefore end in -um.

Holotype:

NHMUK PX TF 312

Fig 3 this study, panels I and J, boring in NHMUK PV R 4205.

Diagnosis:

Boring with chamber diameters ranging from 1.1–2.3 mm and total depths 1.4–2.6 mm. Aperture is fine and straight, with the threadlike aperture neck leading to a chamber that sits at mid-depth beneath the surface of the bone. Chambers have a wide arc and a globular, regular appearance, with symmetry radiating from the upper chamber. Branches are very short and rounded, merging with and almost indistinct from the main chamber body.

Remarks:

This form is unusually globular and regular in shape and is more easily distinguished from other *Osspecus* ichnospecies due to this regularity and the very fine threadlike aperture. Complete borings also tend to preserve due to this geometry. This ichnospecies is found in two marine reptile specimens in this study.

*Osspecus automedon* isp. nov.

LSID urn:lsid:zoobank.org:pub:DD81124D-44C1-41E1-9833-408F7D3AE4E0

Etymology:

After Achilles’s charioteer, due to the thin branches with rounded tips merging at the ends, causing the chambers to have the appearance of a segment of a spoked wheel. The name is used as a noun in apposition without modification.

Holotype:

NHMUK PX TF 313

Fig 2, panels A and B, in Jamison-Todd et al. (2024) [8]. Boring in NHMUK PV R 35103.

Additional examples:

Kiel et al. (2012) [9], Fig 3.

Diagnosis:

Boring with chamber diameters ranging from 0.5–3 mm and depths from 1–5 mm. Aperture neck length is variable, and therefore depth is also variable. Branches are bar-like and remain somewhat separated until they widen into bulbs and merge at the ends. They are very long relative to the chamber diameter, making up almost all of the chamber. The centre of primary radial symmetry sits at the base of the aperture neck. Arc length can be highly variable; cross sections look like sections of a circle.

Remarks:

This ichnospecies was previously recorded in the Chalk Group by Jamison-Todd et al. (2024), alongside two other ichnospecies in one tooth [8]. Borings referred to this ichnospecies were also found in an Oligocene whale tooth by Kiel et al. (2012) [8]. No further examples of this ichnospecies were discovered in the marine reptile specimens presented here. While the morphology is distinct, the length of the aperture neck and the arc length of the total chambers appears to be variable. There may be secondary radial symmetry in the branch ends, causing them to broaden at their tips, but it is not clearly visible.

*Osspecus frumentum* isp. nov.

LSID urn:lsid:zoobank.org:act:DFB1F16F-7AC3-4C2B-BFCA-E7596B7C5CC0

Etymology:

After the Latin for ‘corn’, due to its unique symmetry causing this morphotype to resemble a corncob. The species name is a noun in apposition.

Holotype:

NHMUK PX TF 314

Fig 2, panels E and F in Jamison-Todd et al. (2024) [8]. Boring in NHMUK PV R 35103.

Additional examples:

Higgs et al. (2014) [10], Fig 2, panels M–R.

Diagnosis:

Boring where the chamber consists of a central column connected to a tapering aperture neck that is difficult to distinguish from the chamber. Thin filamentous branches extend laterally from the central column of the chamber. Symmetry is linear, rather than radial, in the vertical section, along the main column of the chamber. In horizontal cross section symmetry would be radial around the columnar chamber.

Remarks:

One chamber was measured at 1 mm diameter and 1.5 mm total depth [16]. This ichnospecies is very easily distinguished from other boring types due to its unique symmetry and resulting distinctive morphology. It occurs in a tooth from the Chalk Group alongside two other ichnospecies [16]. A modern example of this ichnospecies created by *Osedax ryderi* is described by Higgs et al. (2014) [12]. No additional examples are recorded in the present study.

*Osspecus panatlanticum* isp. nov.

LSID urn:lsid:zoobank.org:act:510A40DE-6227–45DE-AC17-0B747E3A9728

Etymology:

This ichnospecies is the most geographically dispersed in the Cretaceous, as it has been found in specimens from Belgium and the Gulf Coastal Plain of North America, and is one of the two ichnospecies occurring on both sides of the Atlantic during this time. For adjectival species names such as this one, we take the secondary poetic usage of the Greek ‘specus’ as neuter, as the original genus was never defined in gender, and Latinized species names therefore end in -um.

Holotype:

IRSNB 7767

Fig 2, panels I and J in Jamison-Todd et al. (2024) [8]. Boring in IRSNB R 370.

Additional examples:

Higgs et al. (2014) [10], Fig 6

Higgs et al. (2010) [11], Fig 1

Diagnosis:

Boring where the aperture neck is short and thin when preserved. Chamber depth is shallow and the chambers roughly hemispherical, with an arc length of approximately 180 degrees. Branches extending beyond the main hemispherical chamber are very thin but rounded at the ends. Primary radial symmetry sits in the centre of the chamber, and there is secondary radial symmetry at the ends of the branches, which form clusters and are irregular in length.

Remarks:

This ichnospecies was called ‘type 5’ in Jamison-Todd et al. (2024), and was found in two mosasaurs from Belgium, and a plesiosaur from North America [8]. Borings measured in Jamison-Todd et al. (2024) are 1–3 mm in diameter and 0.5–1.5 mm in depth [8], reflecting the range of smaller-scale examples and larger pits that may be created by clusters of weathered borings combining into a larger chamber. It was therefore present on both sides of the Atlantic Ocean basin in the Cretaceous, but has not been recorded in the Chalk Group of the United Kingdom. This form is also present in modern whale bones, made by an indeterminate *Osedax* species shown in Higgs et al. (2014) [8], and also the large chambers consisting of overlapping borings created by *Osedax mucofloris* as shown in Higgs et al. (2010) [8]. This ichnospecies is therefore possibly made by two modern species of *Osedax*, though in the former instance the species is unknown, and this could be a case of convergence in boring morphology if this unknown species is not also *Osedax mucofloris*.

In addition, the spelling of the novel species “*Osspecus eunicefootia*” is changed to “*Osspecus eunicefooteae*” in this Correction, for the reasons outlined in the corrected “Systematic ichnotaxonomy” section. All instances of “*Osspecus eunicefootia*” or “*O. eunicefootia*” throughout this study [1] should be “*Osspecus eunicefooteae*” or “*O. eunicefooteae*”, respectively. The authors also provide the updated figure legend for Fig 3 to reflect the amended spelling of “*O. eunicefooteae*”. Please see the corrected Table 2 and Figs 3,4 and 5 containing the updated spelling for this species here.

**Table 2:** The *Osspecus* ichnospecies presented in this study and the characteristic features that were used to define them.

Ichnospecies	Specimens with ichnospecies	Diameter range (mm)	Depth range (mm)	Type diameter (mm)	Type depth (mm)	Type aperture (mm)	Surface penetration	Centre of radial symmetry	Secondary symmetry	Branch length	Branch shape	Chamber shape	Holotype reference	Holotype accession number	In vertebrate specimen
*Osspecus tuscia*	NHMUK PV R 1233; NHMUK PV R 1265; NHMUK PV OR 32812	0.7–3.7	0.6–2.1	Between 1.7 and 3.7	Unknown	Between 0.6 and 1.2	Mid	Aperture neck base	No	Mid	Lobate with pointy ends	Sector	Higgs et al. (2012)	n/a	IGF 1134T
*Osspecus eunicefooteae*	NHMUK PV OR 32812	1.8–2.7	1.2–1.9	3.5	2.7	0.7	Shallow	Top of chamber	No	Long	Wavy and thin with pointed ends	Hemispherical	This study	NHMUK PX TF 309	NHMUK PV OR 32812
*Osspecus morsus*	NHMUK PV R 1215; NHMUK PV R 3355; NHMUK PV R 35103	1.3–2.4	2.3–3.7	1.6	2.5	0.2	Mid to deep	Aperture neck base	Possibly	Short	Lobate with indistinct ends	Sector	This study	NHMUK PX TF 310	NHMUK PV R 1215
*Osspecus campanicum*	NHMUK PV R 1233	0.5–2.4	0.5–2.1	2.4	2.1	0.5	Mid	Upper chamber	Possibly	Mid	Rounded with lobate ends	Irregular rounded	This study	NHMUK PX TF 311	NHMUK PV R 1233
*Osspecus arboreum*	NHMUK PV R 4205; NHMUK PV R 3355	1.1–2.3	1.4–2.6	2	2.2	0.2	Mid	Upper chamber	No	Very short	Rounded	Round	This study	NHMUK PX TF 312	NHMUK PV R 4205
*Osspecus automedon*	NHMUK PV R 35103	0.5–3	1–5	3.1	4	0.6	Mid to deep	Aperture neck base	Possibly	Very long	Spoke with bulb at end	Rounded sector	Jamison-Todd et al. (2024)	NHMUK PX TF 313	NHMUK PV R 35103
*Osspecus frumentum*	NHMUK PV R 35103	single boring	single boring	1	1.5	0.1	Mid	Central axis of chamber	No	Long	Threadlike	Columnar with offshoots	Jamison-Todd et al. (2024)	NHMUK PX TF 314	NHMUK PV R 35103
*Osspecus panatlanticum*	FMNH PR 187; IRSNB 369; IRSNB R 370	1–3	0.5–1.5	0.9	1.3	0.2	Shallow	Centre of chamber	Yes	Short	Narrow with rounded ends	Hemispherical	Jamison-Todd et al. (2024)	IRSNB 7767	IRSNB R 370

**Fig 5:**
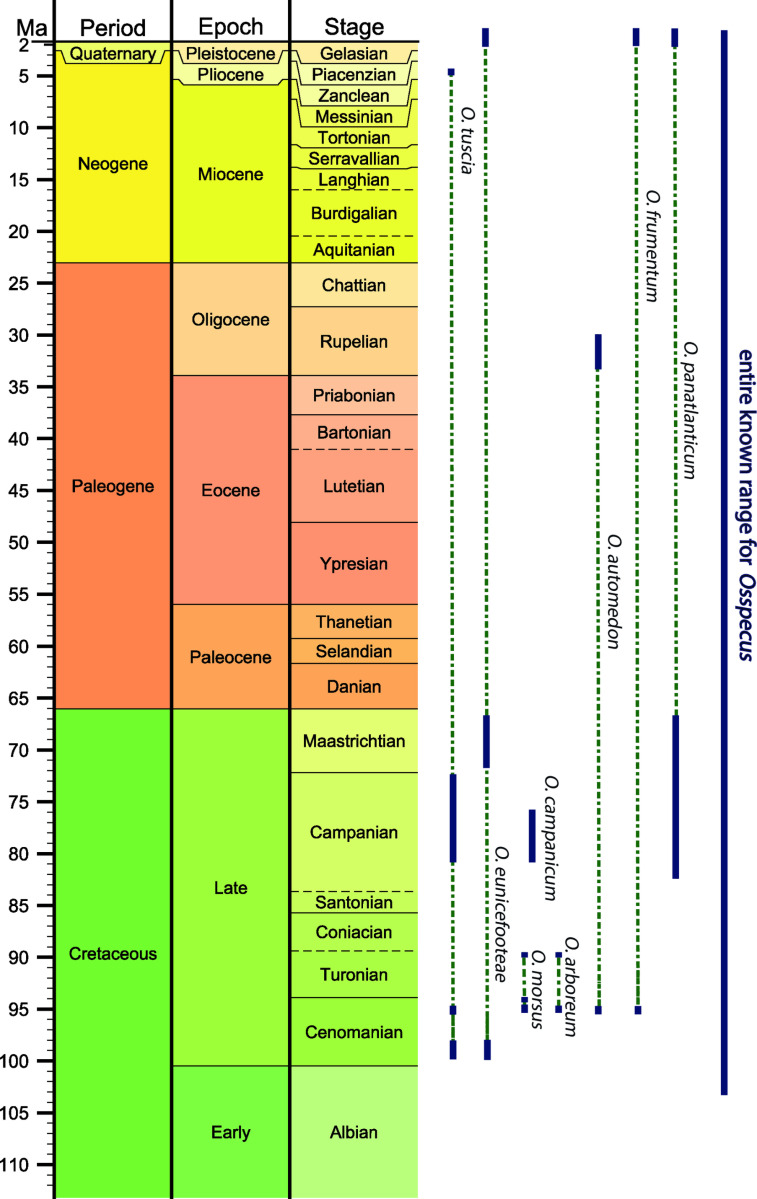
The full time ranges of the eight currently described ichnospecies of *Osspecus.* Earliest fossil occurrence from Danise & Higgs (2015) [15]. Ichnospecies occurrences are indicated by navy bars, and inferred ranges by dashed green lines. Numerical ages from GTS2020 [14] and TimescaleCreator8 (timescalecreator.org).
